# Supplementary material for: Olmesartan Attenuates Kidney Fibrosis in a Murine Model of Alport Syndrome by Suppressing Tubular Expression of TGFβ
Source: Int J Mol Sci. 2019 Aug 6;20(15):3843. doi: 10.3390/ijms20153843 (PMC6695622; doi:10.3390/ijms20153843)
Supplement: Supplementary file 1 [file ijms-20-03843-s001.pdf]

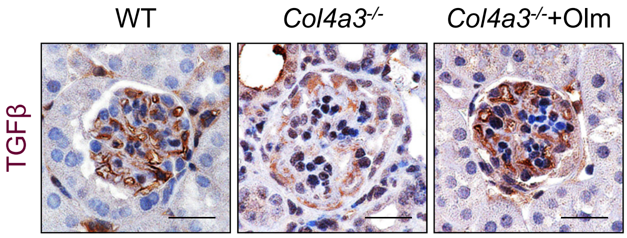

Figure S1. Upregulation of TGFβ is not prominent in the glomerulus of *Col4a3*$^{–/–}$ mice. Representative images of immunohistochemical staining for TGFβ in the glomeruli of WT, *Col4a3*$^{–/–}$, and *Col4a3*$^{-/-}$+Olm mice. Scale bars, 50 μm.

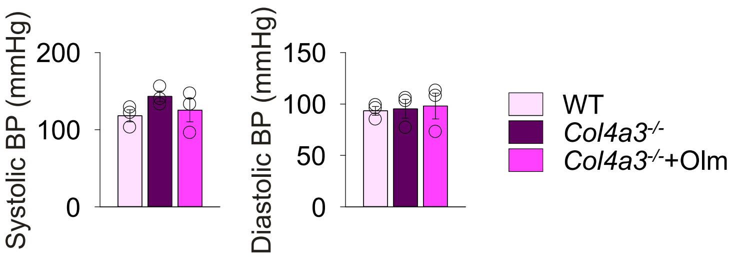

Figure S2. Olmesartan treatment does not affect either systolic or diastolic BPs in the *Col4a3*$^{–/–}$ mice. Comparison of systolic (left) and diastolic (right) BPs measured in the tail of WT, *Col4a3*$^{–/–}$, and *Col4a3*$^{-/-}$+Olm mice (n = 3 mice/group).

Table S1. List of primary and secondary antibodies for immunohistochemistry

	Host	Reactivity	Supplier	Cat. No.
Collagen type 1	Rabbit	Mouse	Abcam	ab34710
F4/80	Rat	Mouse	Bio-rad	MCA497GA
Transforming growth factor β1	Rabbit	Human, Mouse	Abcam	ab92486
α smooth muscle actin	Mouse	Human, Mouse	Sigma-Aldrich	A3854
Rabbit IgG, HRP-linked	Goat	Rabbit IgG	Vector	PI-1000
Rat IgG, HRP-linked	Goat	Rat IgG	Vector	PI-9400
Mouse IgG, HRP-linked	Goat	Mouse IgG	Vector	PI-2000

Table S2. List of primary and secondary antibodies for immunoblotting.

	Host	Reactivity	Supplier	Cat. No.
Angiotensin-converting enzyme	Goat	Human, Mouse	Santa Cruz	sc12187
Angiotensin-converting enzyme 2	Goat	Mouse	R&D	AF3437
Angiotensin-converting enzyme 2	Rabbit	Human	Cell Signaling	#4355
Angiotensin II-III	Mouse	Human, Mouse	Novus Biologicals	NB100-62346
Ang II type 1 receptor	Rabbit	Human, Mouse	Santa Cruz	sc-1173
Ang II type 2 receptor	Rabbit	Human, Mouse	Santa Cruz	sc-9040
BAX	Rabbit	Human, Mouse	Cell signaling	#2772
Bcl-2	Rabbit	Human, Mouse	Cell signaling	#3498
CD68	Rabbit	Mouse	Abcam	ab31630
Caspase 3	Rabbit	Human, Mouse	Cell signaling	#9662
Cleaved caspase 3	Rabbit	Human, Mouse	Cell signaling	#9661
ERK1/2	Rabbit	Human, Mouse	Cell signaling	#9102
Fibronectin	Rabbit	Human, Mouse	Abcam	ab2413
Heme oxygenase 1	Mouse	Mouse	Abcam	ab13248
JNK	Rabbit	Human, Mouse	Cell signaling	#9252
P38	Rabbit	Human, Mouse	Cell signaling	#9212
Phopho-ERK1/2	Rabbit	Human, Mouse	Cell signaling	#9101
Phopho JNK	Rabbit	Human, Mouse	Cell signaling	#9251
Phospho P38	Rabbit	Human, Mouse	Cell signaling	#9215
Phospho SMAD2/3	Rabbit	Human, Mouse	Cell Signaling	#8828
SMAD4	Rabbit	Human, Mouse	Cell Signaling	#38454
SMAD2/3	Rabbit	Human, Mouse	Cell Signaling	#3012
TNFα-converting enzyme (TACE)	Rabbit	Human, Mouse	Millipore	AB19027
Transforming growth factor β	Rabbit	Human, Mouse	Cell Signaling	#3711
α smooth muscle actin	Mouse	Human, Mouse	Sigma-Aldrich	A3854
β-actin	Rabbit	Human, Mouse	Cell Signaling	#3711
Goat IgG, HRP-linked	Rabbit	Goat IgG	Sigma-Aldrich	AP106P
Rabbit IgG, HRP-linked	Goat	Rabbit IgG	Cell Signaling	#7074
Mouse IgG, HRP-linked	Horse	Mouse IgG	Cell Signaling	#7076

Table S3. List of primer sequences for real-time qPCR

	Forward	Reverse
mActa2 (αSMA)	ACTGGGACGACATGGAAAAG	CATCTCCAGAGTCCAGCACA
mCol1a1 (collagen, type I)	GAGCGGAGAGTACTGGATCG	TACTCGAACGGGAATCCATC
mGapdh	TGTGTCCGTCGTGGATCTGA	GATGCCTGCTTCACCACCTT
mIcam1	AACTTTTCAGCTCCGGTCCTG	TCAGTGTGAATTGGACCTGCG
mIl-6	ACAACCACGGCCTTCCCTACTT	CACGATTTCCCAGAGAACATGTG
mFn1 (fibronectin)	ACACGGTTTCCCATTACGCCAT	AATGACCACTGCCAAAGCCCAA
mTgfb1 (TGFβ)	CAACAATTCCTGGCGTTACCTTGG	GAAAGCCCTGTATTCCGTCTCCTT
mTnf (TNFα)	GCATGATCCGCGACGTGGAA	AGATCCATGCCGTTGGCCAG
Vcam1	TCTCTCAGGAAATGCCACCC	CACAGCCAATAGCAGCACAC
